# Reflective functioning skills mediating the relationship between attachment security and therapeutic alliance in mentalization‐based treatment for children

**DOI:** 10.1111/papt.70100

**Published:** 2026-08-19

**Authors:** Pelinsu Bulut Özer, Z. Deniz Aktan, Sibel Halfon

**Affiliations:** ^1^ Department of Psychology Istanbul Bilgi University Istanbul Türkiye; ^2^ Department of Psychology FMV Işık University Istanbul Türkiye

**Keywords:** attachment security, child psychotherapy, early alliance, mentalization‐based treatment, reflective functioning

## Abstract

**Objectives:**

The therapeutic alliance is a critical predictor of psychotherapy outcomes, yet little is known about the child‐specific interpersonal factors influencing its early development in child psychotherapy. This study aimed to examine whether children's pre‐treatment attachment security and reflective functioning (RF) skills predict the strength of early therapeutic alliance.

**Design:**

The study was part of a randomized controlled trial measuring the effectiveness of Mentalization‐Based Treatment for Children (MBT‐C).

**Methods:**

Participants were 71 children aged 7–12 who received the MBT‐C intervention. Pre‐treatment assessments included the Attachment Doll Story Completion Task to measure attachment security, and the Child Reflective Functioning Scale to assess RF skills. Early therapeutic alliance was evaluated at the third week of treatment using the self‐report scale of Therapeutic Alliance Scale for Children (both child and therapist forms).

**Results:**

Results from the mediation model showed that children's reflective functioning skills significantly mediated the relationship between attachment security and early alliance rated by children. Therapist‐rated alliance was found to be insignificant.

**Conclusions:**

Findings highlight reflective functioning as a central mechanism linking attachment security to children's perceptions of therapeutic alliance in MBT‐C. This suggests that children's capacity to understand others' mental states is critical for establishing early alliance.

## INTRODUCTION

Therapeutic alliance refers to the special relationship between therapist and patient in the psychotherapy process. One common conceptualization of alliance is based on Bordin's (1979) work where he held forth a trans‐theoretical definition, independent of specific psychotherapy techniques and interventions. Alliance is a common factor in psychotherapy processes, acting like a glue to hold different elements of treatment together (Bordin, 1979; Gaston, 1990). It has been found strongly linked to patient recovery and outcome (Horvath et al., 2011; Orlinsky et al., 1994). Literature on therapeutic alliance in child psychotherapy is less frequent; however, the findings between alliance and outcome shown in adult psychotherapy have been replicated (Karver et al., 2018; Shirk & Karver, 2003).

### Early therapeutic Alliance

Studies focusing on the relationship between therapeutic alliance and outcome suggest that the timing of alliance measurement may be important, highlighting the effects of early therapeutic alliance (Despland et al., 2001; Horvath & Luborsky, 1993; Sexton et al., 1996). The findings of these studies have shown that alliance measurements taken in the first few sessions predict outcome more strongly than measures taken in the later phases of therapy or than the average measurement of alliance throughout therapy (Andrews et al., 2016; Horvath & Luborsky, 1993; Sexton et al., 1996). In this sense, therapeutic alliance is viewed as a rapport that is instantly developed in the first few sessions (Despland et al., 2001). Similarly, it has been shown that alliance established in the first three sessions changes very little in the later stages of therapy (Eaton et al., 1988). These findings put forth the effect of early therapeutic alliance on outcome and recovery in adult psychotherapy.

Research on early alliance in child and adolescent psychotherapy is relatively new. Hogue et al. (2006) showed that early therapeutic alliance had a significant effect on symptom change with adolescent patients receiving family therapy for substance abuse. Similarly, Cirasola et al. (2022) found that early therapeutic alliance was associated with therapy outcome, predicting a decrease in emotional/behavioural symptoms. Again, in youth psychotherapy, Labouliere et al. (2017) also showed the first‐session therapeutic alliance to be a strong predictor of depressive symptoms in later sessions. For younger aged patients, studies become even more scarce; Chiu et al. (2009) used observer‐rated alliance measures to predict outcome and findings showed that a stronger child‐therapist alliance early in treatment predicted greater improvement in mid‐treatment outcomes. No other studies are known to authors measuring early alliance and its effects on outcome in child psychotherapy settings.

### Factors affecting early therapeutic Alliance

Considering the importance of the relationship between early therapeutic alliance and outcome, the factors that may affect the establishment of early alliance also become vital in psychotherapy research. Literature shows that interpersonal characteristics of patients and therapists may be effective on the strength of early alliance. (Horvath & Greenberg, 1994; Kazdin & Durbin, 2012). Hersoug et al. (2002) argued that quality of past relationships was more significant in predicting alliance than diagnostic characteristics of the patient. In light of this information, insecure relationship schemas may have a consequence of obstructing the initial formation of the alliance relationship. DiGiuseppe et al. (1996) state that ‘children and adolescents have the best chance to form a positive therapeutic alliance if they have a scheme for positive attachments with others, can evaluate their own emotions and behavior, can generate internal attributions for behavior, and believe that their efforts can result in positive outcomes’ (p. 88). In relation to supporting this view, studies show that children with severe externalizing problems (e.g. conduct disorder, antisocial behaviour) have a harder time developing a strong alliance (Kazdin et al., 2005; Zorzella et al., 2015). Children with these destructive tendencies have difficulty in being contained within a relationship, to engage and to reflect on a relationship. These relational patterns impact the formation of early alliance and, considering the strong relationship between early alliance and outcome, may indirectly suggest poorer treatment outcomes. Two of the different interpersonal characteristics are further elaborated; patients' attachment and reflective functioning capacity.

#### Attachment

Attachment experiences can determine the relationship patterns of the person and shape an internal model of relationships (Meyer & Pilkonis, 2001). Studies underlined below have examined the effect of the patient's previous attachment experiences on the formation and maintenance of therapeutic alliance. Kivlighan Jr et al. (1998) found that securely attached patients reported a stronger bond with their therapist as they were able to establish intimacy with the other person more easily. Another study examining the relationship between attachment styles and therapeutic alliance in adult psychotherapies showed that the ability to trust and feel close to others influenced therapeutic alliance positively (Goldman & Anderson, 2007). A meta‐analysis that examined the relationship between attachment styles and alliance conveyed that secure attachment and the strength of the therapeutic alliance was closely associated. Same study also found that alliance reported by the patient had a positive association with attachment security, but alliance reported by the therapist did not have such an effect, marking different informants' attachment styles may be prominent (Diener & Monroe, 2011). While these are adult psychotherapy settings, there are only few studies that highlight the link between attachment and alliance in family psychotherapy settings (Guzder et al., 2011; Johnson et al., 2006). Findings in Guzder et al.'s (2011) study indicate that parents' own attachment security is associated with higher alliance in family therapy, emphasizing the role of parent's general attachment style in forming therapeutic bonds. Johnson et al. (2006) examined the relationship between alliance and parents' attachment to their children. They have found that mother's report of child's attachment security significantly predicted both the mother's and the child's alliance with the therapist. Even though these studies show some work on alliance in family therapy settings, child's own attachment reports are not included.

#### Reflective functioning capacity

Reflective functioning (RF) involves understanding one's own or others' emotions, cognitions, intentions and wishes, accompanied by a verbalization of these mental states. Reflective functioning is closely related to attachment patterns as it involves ‘being in touch’ with the other person's mind. Developmentally, attachment security gets stronger when the parent is more sensitive to the child's states of mind and reflects them. Accordingly, reflective functioning capacity of the child is nurtured from the contact with the primary caregiver and is closely linked with one's relationality (Fonagy et al., 1991).

Reflective functioning is also an essential skill that needs to be developed in therapy; it enables the child to differentiate between subjective experience and the capacity to reflect upon that experience. Still, there is very little research on how reflective functioning comes into play in therapy process research (Ekeblad et al., 2016). In adult psychotherapy, one study has reported that low RF capacity has a negative effect on the formation of early therapeutic alliance (Ekeblad et al., 2016). In another study, RF was shown to be predictive of both the strength of alliance and changes in outcome (Katznelson et al., 2020). Taubner et al. (2011) also showed that higher pretreatment RF was associated with early patient therapeutic alliance and improvement in alliance too. These findings are not yet replicated in child psychotherapy studies, creating a gap in literature.

### Aim of the current study

Building on the evidence that patient‐specific interpersonal characteristics contribute to alliance formation, this study seeks to address a key gap: the role of attachment security and reflective functioning in the early development of alliance in child psychotherapy. While mentalization‐based approaches show promise for improving reflective functioning and attachment security in children (Byrne et al., 2020; Oehlman Forbes et al., 2021), no study has directly investigated whether children's pre‐treatment RF skills mediate the link between attachment and early alliance. The aim of this study was to examine these patient characteristics that may affect the formation of therapeutic alliance in Mentalization‐Based Treatment for Children (MBT‐C; Midgley et al., 2017).

With the purpose of exploring this relationship, we hypothesize that

1. child's pre‐treatment attachment security will be positively associated with children's reflective functioning,
2. child's pre‐treatment reflective functioning skills will be positively associated with the strength of both child and therapist ratings of early therapeutic alliance and
3. children's reflective functioning skills will significantly mediate the relationship between attachment security and the early therapeutic alliance.

## METHOD

The study is conducted within the scope of a randomized controlled trial aiming to test the effectiveness of MBT‐C against a social skills/psychoeducation control group (Halfon et al., 2024). The sample of the current study was chosen from the MBT‐C group of this randomized controlled trial. Firstly, RCT's method will briefly be explained. Later, the current study's method will be detailed.

### Randomized controlled trial

#### RCT sample selection

The recruitment of patients for the randomized controlled trial started in March 2022 and ended in June 2023. Participating patients were selected from families who applied to the university's counselling centre. Inclusion criteria were as follows: (a) children aged between 5 and 12 years and (b) showing clinical level emotional/behavioural problems pre‐treatment as indicated by Child Behavioural Checklist (CBCL; Erol et al., 1995). The exclusion criteria included parental risk of harm to self/others, psychosis, alcohol‐substance abuse as well as children's risk of harm to self/others, psychosis, severe conduct disorder, eating disorders, cognitive impairment or autism spectrum disorders. Ongoing abuse in the family was also referred elsewhere. A total of 222 patients who met these criteria were included in the RCT. Patients were randomly assigned to either the Mentalization‐Based Therapy for Children (MBT‐C) intervention group (*n* = 111) or the social skills/psychoeducation control group (*n* = 111).

#### Mentalization‐based therapy for children (MBT‐C)

Mentalization‐Based Therapy for Children (MBT‐C; Midgley et al., 2017) is a 15‐week psychodynamic and attachment‐based intervention programme aiming primarily to improve the mentalization skills of patients. It is tailored for children between the ages of 5–12, with parallel parent therapy sessions. Mentalization‐based therapies help patients develop better emotional regulation and coping skills by focusing on understanding mental states such as emotions, thoughts and perspectives. Central to MBT‐C is the therapeutic relationship, where therapists maintain a curious, nonjudgmental stance, modelling mentalization to the patient. Specific interventions can be categorized into three main areas: attention control, emotion regulation and explicit mentalization. Attention control involves helping children focus, shift, and regulate their attention through joint attention exercises, mirroring or slowing down interactions to foster engagement. Emotion regulation strategies aim to support children in recognizing and managing their emotional states. Explicit mentalization interventions focus on developing the child's ability to verbalize thoughts and emotions, encouraging them to reflect on their own and others' mental states (Midgley et al., 2017).

In this RCT, the therapists conducting the MBT‐C intervention consisted of 16 therapists who were masters‐level interns and recent graduates of clinical psychology. Their ages ranged between 25 and 41 years (*M* = 29, *SD* = 3.89); 90% of them were women, and experience level ranged between 1 and 6 years (*M* = 3.07, *SD* = 1.66). The therapists received standard MBT‐C training (6 sessions of 3 h in total) given by official trainers and received supervision from the same experts for the therapies they conducted.

#### Ethical approval

The project titled ‘Effectiveness of Mentalization‐Based Therapy for Children (MBT‐C) and Change Mechanisms’ received ethical approval prior to start of the study (no: 2021–40,024‐48). Written informed consent was obtained from the parents, and verbal consent was obtained from children. The data collected within the scope of the study were kept in accordance with the principles of confidentiality.

### Current study

#### Participants

For the current study's sample, children who were allocated to the MBT‐C intervention were selected. Out of the 111 patients who were allocated to the MBT‐C arm, our study included patients whose age ranged between 7 and 12 years. We excluded younger children due to age limits of the self‐report therapeutic alliance scale used in this study. A priori power analysis was also conducted using G*Power to determine the appropriate sample size for linear multiple regression assuming *α* = .05, power = .80, *f* ^2^ = 0.15. A sample size of 68 was estimated. Seventy‐five patients who met the age inclusion criteria were included; however, four of these patients dropped out during the assessment phase; analyses were conducted with the remaining 71 patients. Demographic information of these children and their parents is presented in Table 1.

**TABLE 1:** Socio‐demographic characteristics of patients included in the study (*N* = 71).

	*n* (*%*)	*M* (*SD*)
Child age		8.92 (1.60)
7–8	35 (49)	
9–10	22 (31)	
11–12	14 (20)	
Mother age		38.07 (4.65)
Father age		41.21 (4.84)
Child sex		
Female	26 (36)	
Male	45 (63)	
Child Pre‐treatment problem type		
Internalizing problems	31 (44)	
Externalizing problems	11 (15)	
Comorbid problems	29 (41)	
Parent marital status		
Married	57 (80)	
Divorced/separated	14 (20)	
Family socio‐economic status		
Low	17 (24)	
Mid‐low	13 (18.3)	
Middle	13 (18.3)	
Mid‐high	15 (21)	
High	13 (18.3)	
Total session participated		12.08

*Note*: Child pre‐treatment problem type was measured by Child Behaviour Checklist (CBCL). CBCL was gathered from parents at pre‐treatment for determining inclusion criteria of showing clinical level emotional/behavioural problems. Internalizing problems include anxiety, depressive problems, somatic concerns. Externalizing problems include behavioural problems, hyperactivity and problems related to impulse control. Comorbid problems include symptoms from both categories. Clinical cut‐off scores were determined by CBCL; standardized T scores of 65 and above were considered clinical level. Family socio‐economic status was determined based on income relative to the minimum wage of the year that the study was conducted.

#### Data collection tools

##### Sociodemographic information form

The form included basic information about socio‐demographic characteristics of participants such as child's age, gender, family SES and marital status.

##### Therapeutic Alliance scale for children child and therapist forms (TASC)

The Therapeutic Alliance Scale for Children (Shirk et al., 2011; Shirk & Saiz, 1992) is a self‐report scale designed to assess aspects of therapeutic alliance in child psychotherapy. There are three versions of TASC: child form, parent form and therapist form. Original TASC contains 12 items that are scored on a 4‐point Likert scale ranging from 1 (‘*strongly disagree*’) to 4 (‘*strongly agree*’). Higher scores indicate a more positive/stronger therapeutic alliance. Original scale revealed a three‐factor structure (positive bond, negative bond and verbalization of therapy goals) with each subscale showing adequate consistency. The total internal consistency of the TASC child form was found to be .71, whereas the therapist form revealed an internal consistency score of .82. Subsequent psychometric studies of TASC show a two‐factor structure; positive and negative therapeutic alliance (Accurso & Garland, 2015; Creed & Kendall, 2005; Fjermestad, 2012; Hawley & Weisz, 2005; Kazdin et al., 2005).

TASC child and therapist forms were translated to Turkish and psychometric properties were investigated. This study also put forth a two‐factor structure similar to the subsequent psychometric studies of the scale. Items that constituted the Negative Alliance factor (items 2, 5, 8, 11) were reverse coded to gather a total score. The internal consistency reliability scores of TASC subscales and total scores were moderate to excellent (.65–.91) in both child and therapist forms. The Turkish version of TASC also showed adequate validity scores. (Bulut Özer, 2023).

In the current study, we used TASC child and therapist forms; they show an internal consistency score of .81 and .92 respectively.

##### Attachment doll story completion task (ASCT)

First developed by Granot and Mayseless (2001) for assessing attachment in middle childhood, this task is a semi‐structured play procedure that is used to assess the quality of relationship with caregivers. The scale includes five stories in total: stories initiated by the practitioner using various materials (human figures, toys) and the child is asked to complete the stories. The themes of the stories elicit the attachment patterns of the child. Criteria considered when rating attachment security are; open expression of feelings, the nature of the parent–child relationship (e.g. the level of sensitivity and reactivity of the parent), the positive resolution of the conflict and the child's consistency with the theme of the story.

Child's attachment style was rated on categories of secure, anxious, avoidant and disorganized types on a 5‐point Likert scale (1 = *does not reflect this attachment type at all*; 5 = *completely reflects this type of attachment*). Later, the total attachment security of the entire protocol was also calculated: 1 (*completely similar to insecure attachment; 3 or more stories are rated as insecure*), 2 (*partially insecure attachment; 1 or 2 stories are rated as insecure*) and 3 (*completely similar to secure attachment; all 5 stories rated as secure*). There was a good agreement between raters for the total attachment scores (80% agreement, kappa = .68); in addition, there was adequate reliability for each of the prototypes (secure *r* = .80, avoidant *r* = .78, anxious *r* = .82, disorganized *r* = .85). The scale was adapted to Turkish by Uluç (2005) with a good overall inter‐rater reliability score (kappa = .83), and for each of the stories inter‐rater reliability ranged between .81 and 1.

In the current study, four pilot protocols were coded by eight assistants. Six assistants with acceptable reliability coefficients (min ICC = .70) were asked to code 10 more pilot tests, and inter‐rater agreement results were calculated. All coders reached a sufficient ICC (*M* = 0.89, *SD* = 0.07). The main data after the training were coded in pairs and inter‐rater reliability was again calculated (range = .77–1; ICC *M* = .93, *SD* = 6.48).

##### Child reflective functioning scale (CRF)

Child Reflective Functioning Coding measures the reflective functioning capacity of children over the age of 7 years in the attachment environment (Ensink et al., 2013, 2015). It is assessed through rating the narrative of the child's attachment interview (Child Attachment Interview, Shmueli‐Goetz et al., 2008). The Child Attachment Interview includes questions about attachment relationships, feelings, needs and attributions. The raters score each answer in the attachment interview on a scale from −1 to 9 (−1 = *Negative reflective functioning*, 5 = *Ordinary reflective functioning*, 9 = *Full/extraordinary reflective functioning*). Lower scores indicate evasion of mentalizing, criticizing, becoming silent, bizarre explanations; middle scores refer to mental states that are not presented in detail and generally reflect simple mentalizing capacity; higher scores indicate descriptive and elaborative mentalizing capacity, generally showing a multilayered structure or a causal sequence. The total score of reflective functioning constitutes two parts: reflective functioning skills about self and reflective functioning skills about others. The sum of these two constructs generates a total CRF score. In the original study, CRF items showed high internal consistency (*α* = .94) and total score showed good inter‐rater reliability (ICC = .93) (Ensink et al., 2015).

For the current study, six pilot Child Attachment Interviews were coded for RF by four assistants in the training phase. All coders reached sufficient intraclass correlation coefficient (*M* = .86, *SD* = 0.01) during training. The main data were coded in pairs and inter‐rater reliability values were calculated (range = .78–1; ICC *M* = .90, *SD* = 7.71).

### Procedure

In a pretreatment assessment, Attachment Doll Story Completion Task and Child Attachment Interview were applied to children in order to measure their attachment security and reflective functioning skills. This assessment was recorded for coding purposes. Therapeutic alliance measurements were collected from both children and therapists at the third week of treatment (after the last session of the assessment process) to evaluate the strength of early alliance. Both TASC forms (therapist and child) were collected through online survey software. To facilitate the process, an assistant reached out to the parents of the child patient to schedule a brief interview with the child to help fill out the alliance scale independently.

### Data analytic strategy

The hypotheses were tested using the PROCESS macro for SPSS (Hayes, 2022). Model 4 was employed to examine whether children's reflective functioning (CRF‐Self and CRF‐Other) mediated the relationship between attachment security and the therapeutic alliance. In accordance with statistical recommendations, the indirect effects were evaluated using non‐parametric bootstrapping with 10,000 resamples. Mediation is considered significant if the 95% bias‐corrected confidence interval (CI) does not include zero. Separate parallel mediation models were conducted for child‐rated and therapist‐rated alliance to determine if the mediating roles of self‐ and other‐directed reflective functioning skills are significant. All analyses were conducted using SPSS v28.

## RESULTS

Prior to conducting the PROCESS macro analyses, assumptions of ordinary least squares regression were examined. Linearity, homoscedasticity, independence of errors and multicollinearity were assessed and found to be met. To address any potential violations of normality in the indirect effect, 10,000 bootstrap resamples were utilized. Additionally, outliers were evaluated using standardized residuals and Cook's distance values; there were no outliers and all the data were retained.

The descriptive statistics for all variables are presented in Table 2. Specific types of attachment scores were not included in the regression analyses, especially with the disorganized type falling out of the proposed scores of the normal distribution due to low variance. Instead, attachment security total score was used to best reflect the attachment security level of the child.

**TABLE 2:** Descriptives of pre‐treatment attachment security, CRF ratings and early alliance ratings.

	*M*	*SD*	Range	Skewness	Kurtosis
CRF self	2.05	.96	0–5	.07	.69
CRF other	2.26	.89	0–5	.13	.31
CRF total	2.21	.87	0–5	.06	.62
Secure attachment	2.44	.97	1–4.5	.36	−.82
Anxious attachment	2.66	1.18	1–5	.11	−1.22
Avoidant attachment	2.35	1.32	1–5	.59	−1.14
Disorganized attachment	1.32	.85	1–5	3.07	9.06
Attachment security total score	1.35	.58	1–3	1.5	1.18
TASC child	35.21	4.48	23–44	−.55	.49
TASC therapist	30.59	6.82	17–44	−.19	−.78

Abbreviations: CRF other, child reflective functioning skills about others; CRF self, child reflective functioning skills about self; CRF total, child reflective functioning total score; TASC, therapeutic alliance for children.

Correlation analysis was conducted between all variables that will be in the mediation model. The results of this analysis (presented in Table 3) show that Attachment Security Total Score is significantly and positively correlated with CRF Self and Other variables. There is also a significant association between Attachment Security Total Score and Therapeutic Alliance scores of both the child and the therapist. In addition, CRF Other is only significantly associated with TASC Child but not with TASC Therapist scores, whereas CRF Self scores are moderately but significantly associated with both alliance scores.

**TABLE 3:** Correlations between pre‐treatment attachment security, CRF ratings and early alliance ratings.

	1	2	3	4	5
1. CRF self	–				
2. CRF other	.64**	–			
3. Attachment security total score	.21*	.33*	–		
4. TASC child	.26*	.34*	.26*	–	
5. TASC therapist	.28*	.13	.27*	.37**	–

Abbreviations: CRF other, child reflective functioning skills about others; CRF self, child reflective functioning skills about self; TASC, therapeutic alliance for children (child and therapist forms).

* *p* < .05;

** *p* < .01.

To account for the multicategorical nature of the covariate Problem Type (Internalizing, Externalizing, Comorbid), the variable was dummy‐coded before proceeding with mediation analyses. The Internalizing group was used as the reference category, and two indicator variables (Externalizing vs Internalizing and Cormorbid vs Internalizing) were included as covariates in the mediation model.

In order to test the hypotheses, two separate parallel mediation models were run with three control variables: age, gender and problem type. In Model 1, attachment security was entered in the model as IV, CRF Self and Other scores were entered as mediators, and child‐rated alliance scores were DV. In Model 2, therapist‐rated alliance scores were entered as DV; IV and mediators remained the same.

In Model 1, the total effect of attachment security on child‐rated alliance was significant (*B* = 1.68, *SE* = .89, *p* = .05). Results revealed that attachment security significantly predicted CRF‐Other (Path *a*: *B* = .22, *SE* = .07, *p* = .02), which in turn significantly predicted child‐rated alliance (Path *b*: *B* = .88, *SE* = .33, *p* = .03). As for the indirect effect, the pathway through CRF‐Other was significant (*B* = .20, 95% CI [0.14, 0.27]). Conversely, the pathway through CRF‐Self was not significant (*B* = .06, 95% CI [−.20, 1.05]). Notably, the direct effect of attachment on alliance became insignificant (*c′*: *B* = 1.61, *p* = .09) upon the inclusion of the mediators, indicating a pattern of full mediation through the child's ability to reflect on others. The final model explained 21% of the variance in child‐rated alliance (*R* ^*2*^ = .21, *F* = 2.22, *p* = .05). Mediation path is presented in Figure 1.

**FIGURE 1:**
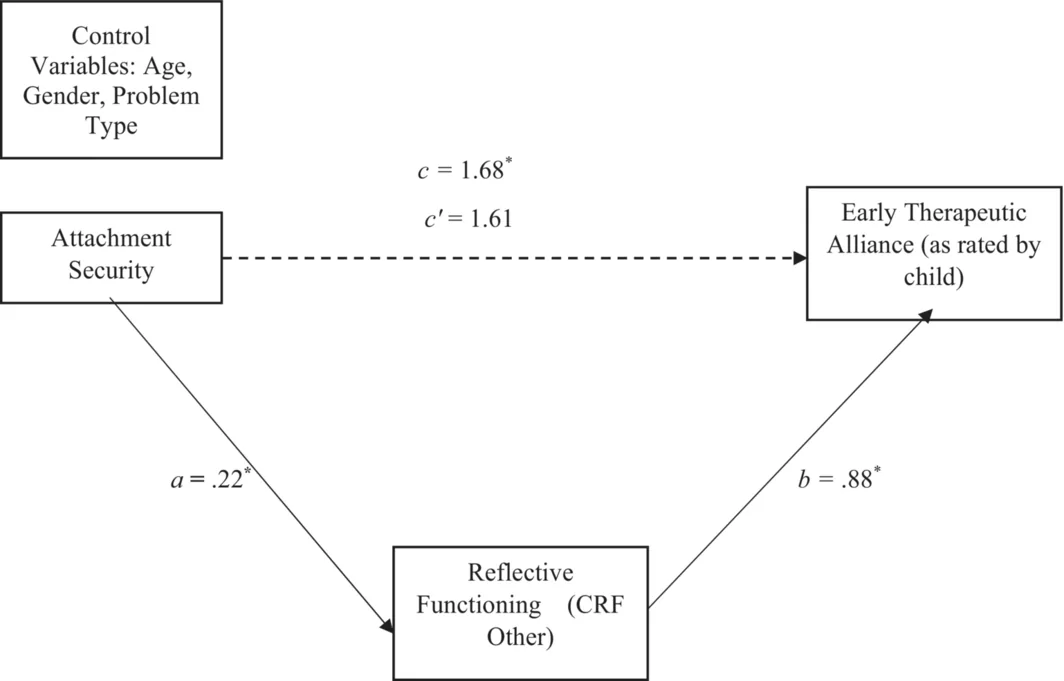
Mediation model of attachment security and child‐rated alliance via reflective functioning. CRF other, child reflective functioning skills about others. Figure shows unstandardized coefficient.

In Model 2, the total effect of attachment security on therapist‐rated alliance was also significant (*B* = 3.18, *SE* = 1.38, *p* = .02). However, unlike the child model, the indirect effects through both CRF‐Self (*B* = .52, 95% CI [−.15, 1.85]) and CRF‐Other (*B* = .01, 95% CI [−.67, .30]) were non‐significant, as their 95% confidence intervals both included zero. The direct effect of attachment security remained significant (*c′*: *B* = 2.62, *p* = .02), indicating that while attachment predicts the therapist's perception of the alliance, this relationship is not mediated by the child's reflective functioning. Table 4 presents the results of mediation analyses.

**TABLE 4:** Mediation results for attachment security on child and therapist‐rated alliance via reflective functioning.

	*B*	*SE*	*t*	*p*	95% CI	
					*LL*	*UL*
Model 1: Child‐rated alliance						
Total effect (*c*)	1.68	0.89	1.88	.05*		
Path a (CRF‐other)	0.22	0.07	3.14	.02*		
Path b (CRF‐other)	0.88	0.33	2.66	.03*		
Direct effect (*c′*)	1.61	0.95	1.69	.09		
Indirect effects (bootstrapped)						
Attachment → CRF‐other → Alliance	0.20	0.03	–	–	**0.14**	**0.27**
Attachment → CRF‐self → Alliance	0.06	0.34	–	–	−0.20	1.05
Model 2: Therapist‐rated alliance						
Total effect (*c*)	3.18	1.38	2.30	.02*		
Path a (CRF‐other)	0.22	0.07	3.14	.02*		
Path b (CRF‐other)	0.01	0.30	0.03	.90		
Direct effect (*c′*)	2.62	1.11	2.36	.02*		
Indirect effects (bootstrapped)						
Attachment → CRF‐other → Alliance	0.01	0.24	–	–	−0.67	0.30
Attachment → CRF‐self → Alliance	0.52	0.52	–	–	−0.15	1.85

*Note*: Confidence intervals for indirect effects were calculated using 10,000 bootstrap resamples. Covariates (age, gender and problem type) were included in all models but are not shown for parsimony. Bold text indicates a statistically significant indirect effect (95% CI does not include zero).

Abbreviations: *B*, unstandardized coefficient; *SE*, standard error.

* *p* < .05.

Regarding the covariates, child's age was a marginal predictor of the alliance in Model 1 (*B* = .68, *p* = .06), indicating a trend where older children reported slightly higher alliance scores. However, age was not a significant predictor in Model 2 (*p* = .44) where therapist‐rated alliance was tested. Other covariates (gender and problem type) were also insignificant across both models (*p* values ranging from .28 to .92).

To sum up the findings, there is partial evidence for our hypotheses. While full mediation was observed for child‐rated alliance, the relationship between attachment and therapist‐rated alliance remained direct. Furthermore, while we hypothesized that both dimensions of reflective functioning would serve as mediators, the results specifically highlighted the role of other‐directed reflective functioning as the primary mechanism linking attachment to the child's perception of alliance.

## DISCUSSION

We have investigated the extent to which the child's attachment security and reflective functioning skills predict the early therapeutic alliance measurement. The results of the mediation analysis partially supported our hypotheses. The predictive effect of attachment security disappeared after reflective functioning skills were assigned to the model and a mediation effect of other‐directed reflective functioning was significant in predicting child's alliance ratings. Results also showed that attachment security and reflective functioning did not predict the alliance measurements taken from the therapist.

Literature conveys that interpersonal relationship characteristics of the patients may be associated with therapeutic alliance (Kazdin & Durbin, 2012). The findings of this study also point to the fact that reflective functioning skills develop within the framework of the attachment relationship: the development of self as an interpersonal agent emerges as children experience themselves in relationships with others. Attachment theory suggests that repetitive experiences of relationships between the self and the other form the child's internal representations in an abstract way and form a schema for subsequent relationships (Fonagy & Target, 1997). In secure attachment experiences, the child can self‐regulate through the caregiver's ability to ‘contain’ the child in response to negative emotions and to provide care both physically and emotionally. The caregiver's ‘containment’ skill here also brings along reflective functioning skills such as thinking about mental processes and verbalizing them. In insecure attachment experiences, these skills are disrupted: avoidant caregivers may have difficulty understanding and reflecting on the child, while anxious caregivers may be too busy with their own mental processes (Fonagy & Target, 1997). The empirical studies examining the relationship between attachment and reflective functioning skills also show significant results (Fonagy et al., 1995; Slade et al., 2005). Slade et al. (2005) found that mothers with a secure attachment pattern showed higher reflective functioning skills. The same study also showed a significant reflective functioning capacity of the mother mediated mother's own attachment security and the child's attachment security (Slade et al., 2005). Findings from another study show that mothers with high reflective functioning skills are more securely attached, more independent and their children's attachment patterns at age 1 also reflect secure attachment too (Fonagy et al., 1991, 1995). Other studies show that adolescents with high reflective functioning show higher attachment security characteristics compared to others with low reflective functioning (Bouchard et al., 2008; Fonagy et al., 1991).

Moreover, Nazzaro et al. (2017) found that reflective functioning fully mediated the relationship between attachment and psychological characteristics in an adult clinical population. Beeney et al. (2015) showed the mediating effect of reflective functioning between attachment security and antisocial characteristics. Another study presented evidence towards the mediation effect of reflective functioning between parents' attachment security and parenting attitudes (Nijssens et al., 2018). These studies convey a close‐knit association between attachment and reflective functioning capacity in both child‐rearing and clinical settings.

The results of this study also mark a difference between patient and therapist reports of alliance; the therapist ratings were not significant. In a meta‐analysis examining the relationship between adult attachment security and therapeutic alliance, it was shown that alliance measurements taken from the patient were more significantly related to attachment security than the therapist measurements (Diener & Monroe, 2011). In another study by Diener et al. (2009), positive and significant results were found between adult attachment style and therapeutic alliance, but it was emphasized that these alliance measurements were only self‐reports taken from the patient. These results may point out the fact that patients may be evaluating the therapy relationship according to their own interpersonal characteristics and responding in such a fashion whereas therapists are better at discerning their attachment patterns from their alliance relationship. In order to better understand multi‐informant differences, factoring in the attachment style and interpersonal characteristics of the therapist would also yield rich results.

The marginally significant effect of age may align with developmental perspectives, suggesting that older children can establish a higher level of alliance. The findings of previous studies examining age and therapeutic alliance provide mixed results. While some studies have found that the effect of age on the therapeutic alliance is not significant (Chu et al., 2014; Karver et al., 2018), other studies have shown otherwise (McLeod, 2011). In our study, we did not have a wide age range and since younger children or adolescents were not included, it is difficult to fully determine the effect of age. However, considering the findings, the fact that older children showed higher alliance strength may mean that abstract thinking and higher level cognitive skills may contribute to their interpersonal dialogue. Other studies that focus on age and alliance also point out children's abstract thinking skills and ability to make sense of problems (Graves et al., 2017).

Moreover, the result regarding the total effect of attachment security on alliance is also consistent with the literature; there is a positive and significant relationship between secure attachment and alliance strength. Diener and Monroe (2011) found in their meta‐analysis examining the relationship between attachment security and therapeutic alliance that individuals with high attachment security reported higher alliance measurements. Smith et al. (2010) also replicated the results between secure attachment and therapeutic alliance. With these findings in hand, Harris (2004) states that therapists should be aware of their patients' attachment style and manage the alliance relationship accordingly.

In addition, the reflective functioning variable in the model consisted of two separate variables as CRF Self (reflective functioning skills directed towards one's own mental processes) and CRF Other (reflective functioning skills directed towards the other person's mental processes). Of these variables, only the CRF Other variable was found to have significant results. There are very few studies examining the difference between the two different subcategories of reflective functioning and these are generally in child‐rearing settings rather than therapeutic settings. In one study by Halfon and Besiroglu (2021), other (child‐focused) reflective functioning skills by the parent were predictive of treatment outcome. Suchman et al. (2010) examined the reflective functioning of parents separately for self and other (child) and found that reflective functioning skills for others shape more communicative bids and responses to behavioural cues. It is possible that reflective functioning processes for others are more prominent in the relational field and therefore find more space in the therapy relationship too.

In addition, CRF Self also shows rather low variance in our data. This may also have a disadvantageous effect on significance. A larger sample size may be needed in order to better measure the effect of this variable. It is also widely reported in the literature that reflective functioning skills are lower in clinical groups (Slade, 2005); therefore, it would be useful to expand the sample diversity to non‐clinical groups for a high variance on reflective functioning scores.

A limitation of the study is about the power analysis conducted; G*Power analysis is not designed to account for indirect effects in mediation models. Though the regression analyses examining direct associations were adequately powered, it is worth noting that a Monte Carlo simulation approach may have better suited to estimate power for mediation analysis. Therefore, the study may have been underpowered to detect indirect effects and results of the mediation hypothesis should be interpreted with this limitation in mind. Replication with a larger, adequately powered sample is needed before conclusions can be made about this mechanism.

Another important methodological limitation of the current study is the cross sectional nature of the pre‐treatment variables. More specifically, children's attachment security and reflective functioning were measured concurrently at baseline; however, early therapeutic alliance ratings were gathered around the third session. Though alliance ratings have some temporal separation, the relationship between attachment and reflective functioning does not assume temporal precedence. This may impede the results from being statistically reflective of a causal precedence. Consequently, it may not be possible to rule out reverse pathways between X and M (e.g. higher reflective functioning capacity shaping the attachment representations reported at baseline), or the influence of some unmeasured confounding variables (e.g. parental reflective functioning, child's verbal ability etc.) that may affect both pre‐treatment variables. Future studies can include longitudinal designs that track these variables at separate intervals in the initial phase of therapy to test the directionality in this model.

Overall, the results are novel to the literature in a few ways. This is one of the first studies to examine the effects of attachment security and reflective functioning together on alliance. Moreover, differences between patient and therapist alliance in child psychotherapy are not widely studied. The current study has put forth findings that are important for the gaps in this literature. Gathering more evidence on how pre‐treatment attachment‐related concepts come into play in the therapy relationship would be beneficial for therapists to better approach alliance issues and shape interventions. Thus, findings are efficient in supporting how therapeutic alliance should be treated in child psychotherapy processes.

## AUTHOR CONTRIBUTIONS

**Z. Deniz Aktan:** Writing – review and editing. **Sibel Halfon:** Writing – review and editing. **Pelinsu Bulut Özer:** Conceptualization; formal analysis; methodology; writing – original draft.

## CONFLICT OF INTEREST STATEMENT

The authors hereby declare that they have no conflict of interest.

## Data Availability

The data are not publicly available due to privacy restrictions.
